# Supplementary material for: Long-Term Antibacterial Effect of Electrospun Polyvinyl Alcohol/Polyacrylate Sodium Nanofiber Containing Nisin-Loaded Nanoparticles
Source: Nanomaterials (Basel). 2020 Sep 10;10(9):1803. doi: 10.3390/nano10091803 (PMC7559420; doi:10.3390/nano10091803)
Supplement: Supplementary file 1 [file nanomaterials-10-01803-s001.pdf]

nanomaterials

Article

Long-Term Antibacterial Effect of Electrospun Polyvinyl Alcohol/Polyacrylate Sodium Nanofiber Containing Nisin-Loaded Nanoparticles

Yue Jiang [1,†], Donghui Ma [1,†], Tengteng Ji [1], Dur E Sameen [1], Saeed Ahmed [1], Suqing Li [1,*] and Yaowen Liu [1,2,*]

1 College of Food Science, Sichuan Agricultural University, Yaan 625014, China; jiangyue@stu.sicau.edu.cn (Y.J.); 18894314357@163.com (D.M.); jtt5881@163.com (T.T.J.); sameen0388@gmail.com (D.E.S.); saeedahmedM1993@gmail.com (S.A.)
2 School of Materials Science and Engineering, Southwest Jiaotong University, Chengdu 610031, China
* Correspondence: lsq03_2001@163.com (S.L.); lyw@my.swjtu.edu.cn (Y.L.); Tel.: +86-835-8763-4068 (S.L. and Y.L.); Fax: +86-835-8763-4069 (Y.L.)
† These authors contributed equally to this work

Table S1. Actual levels at coded factor levels of independent variables used in the RSM.

Symbol	Independent variable	Actual levels at coded factor levels		
		-1	0	1
X_1	pH	3	4	5
X_2	Ultrasonic time (min)	0	2.5	5
X_3	E_1/E_2	0.43	1.38	2.33

Table S2. Experimental designs and results of the response surface methodology.

Samples	Factor X_1	Factor X_2	Factor X_3	EE %	
	E_1/E_2	pH	UT min	Actual Value	Predicted Value
1	-1	0	-1	78.00	77.78
2	1	-1	0	69.4	69.28
3	0	0	0	84.38	85.41
4	-1	-1	0	69.60	69.86
5	0	-1	-1	67.20	67.17
6	1	0	-1	81.10	81.25
7	0	1	-1	74.64	74.75
8	0	0	0	85.00	85.41
9	0	0	0	85.58	85.41
10	0	-1	1	70.81	70.71
11	1	1	0	83.00	82.74
12	0	0	0	86.51	85.41
13	-1	0	1	83.9	83.75
14	0	1	1	82.52	82.44
15	0	0	0	85.60	85.41
16	1	0	1	84.40	85.12
17	-1	1	0	75.7	75.82

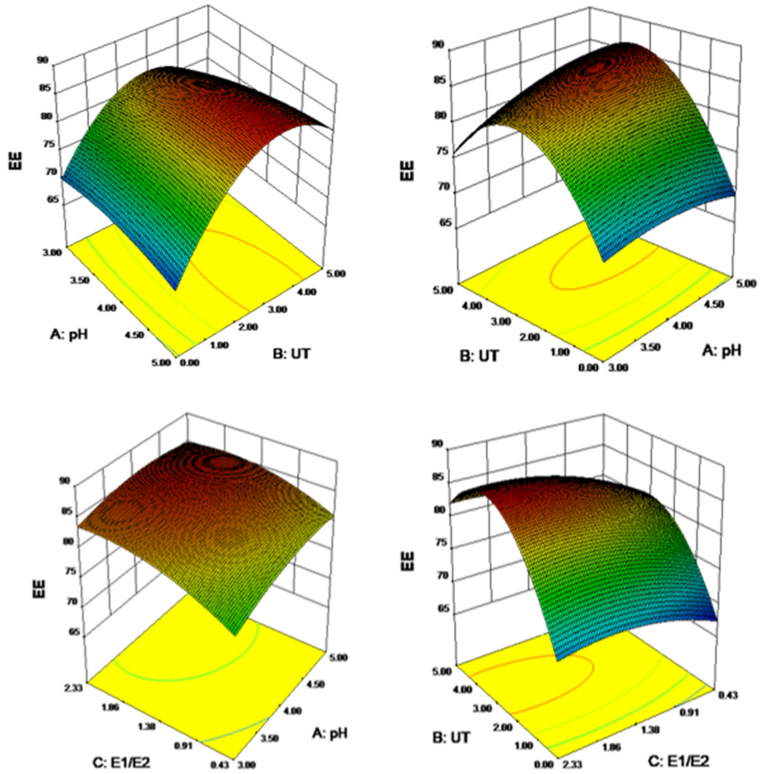

Figure S1. Response surface plots and contour plots showing the effects of pH (X_1), ultrasonic time(X_2) and E_1/E_2 (X_3) on and encapsulation efficiency and interactions between them.

© 2020 by the authors. Licensee MDPI, Basel, Switzerland. This article is an open access article distributed under the terms and conditions of the Creative Commons Attribution (CC BY) license (http://creativecommons.org/licenses/by/4.0/).
